# Strengthening Mechanism of Rotary-Forged Deformable Biodegradable Zn-0.45Li Alloys

**DOI:** 10.3390/ma16083003

**Published:** 2023-04-10

**Authors:** Feng Ding, Yi Zhang, Xinglong Zhu, Pushan Guo, Lijing Yang, Qingke Zhang, Cheng Xu, Wensheng Sun, Zhenlun Song

**Affiliations:** 1 Key Laboratory of Marine Materials and Related Technologies, Zhejiang Key Laboratory of Marine Materials and Protective Technologies, Ningbo Institute of Materials Technology and Engineering, Chinese Academy of Sciences, Ningbo 315201, China; 2 School of Mechanical Engineering and Rail Transit, Changzhou University, Changzhou 213164, China; 3 Ningbo Power Way Alloy Material Co., Ltd., Ningbo 315145, China

**Keywords:** Zn-0.45Li alloy, rotary forging deformation, microstructural, mechanical properties, biodegradable

## Abstract

The use of zinc (Zn) alloys as a biodegradable metal for medical purposes has been a popular research topic. This study investigated the strengthening mechanism of Zn alloys to enhance their mechanical properties. Three Zn-0.45Li (wt.%) alloys with different deformation amounts were prepared by rotary forging deformation. Their mechanical properties and microstructures were tested. A simultaneous increase in strength and ductility was observed in the Zn-0.45Li alloys. Grain refinement occurred when the rotary forging deformation reached 75.7%. The surface average grain size reached 1.19 ± 0.31 μm, and the grain size was uniformly distributed. Meanwhile, the maximum elongation of the deformed Zn-0.45Li was 139.2 ± 18.6%, and the ultimate tensile strength reached 426.1 ± 4.7 MPa. In situ tensile tests showed that the reinforced alloys still broke from the grain boundary. Continuous and discontinuous dynamic recrystallization during severe plastic deformation produced many recrystallized grains. During deformation, the dislocation density of the alloy first increased and then decreased, and the texture strength of the (0001) direction increased with deformation. Analysis of the mechanism of alloy strengthening showed that the strength and plasticity enhancement of Zn-Li alloys after macro deformation was a combination of dislocation strengthening, weave strengthening, and grain refinement rather than only fine-grain strengthening as observed in conventional macro-deformed Zn alloys.

## 1. Introduction

Zinc (Zn) is a nutrient for developing the immune organ thymus. Only adequate Zn intake can effectively ensure thymus development, normal differentiation of T lymphocytes, and promote cellular immune function [1]. Zn meets the safe range of metal ions that can be absorbed by the human body and gradually corrodes medical materials. As a medical degradable metal, Zn and its alloys have a more suitable degradation rate than magnesium- and Ferume-based alloys. Zn also has good biocompatibility [2,3,4,5]. Therefore, Zn alloys are currently used in biodegradable implants, such as vascular stents [6,7], gastrointestinal anastomosis staples [8], and bone plates [9]. However, Zn’s mechanical properties and machinability are relatively poor due to its crystalline structure (hexagonal close-packed) [4,10]. Current research is dedicated to improving the mechanical properties of Zn alloys, which is usually realized by adding different elements to Zn alloy via casting to create binary and multiple alloys such as Zn-Mn [2], Zn-Cu [11], and Zn-Mg [12]. Among all the elements, lithium (Li) is particularly effective in increasing the strength of Zn alloys; the strength of Zn-Li alloys is comparable with that of pure titanium and stainless steel [13,14]. Zhao et al. [15] found that the yield strength of Zn alloy can be increased by 2–2.5 times by adding 0.1 wt.% Li to the medium pure Zn. Sun et al. [16] confirmed in the experiment that the mechanical properties of Zn-0.5Li alloy rib plate are comparable with those of pure titanium. Li is also one of the elements required for human health. It regulates the central nervous system and strengthens the body’s resistance. However, the cast plasticity of cast Zn-Li alloys is poor at room temperature [14]. Alloys with low plasticity and poor processability cannot be modified for processing as medical implant devices.

The most common method to improve the plasticity of alloys is grain refinement. Huang et al. [17] fabricated fine-grained Zn-Al alloys by Zn-Al master alloy and hot rolling. Fine-grained Zn-Al alloys have good mechanical strength and corrosion rate. Wiktor et al. [18] prepared Zn alloys with micron-sized grains through equal channel angular pressing (ECAP) of Zn alloys with the addition of Ag, Cu, and Mn (0.5 at.%) to the Zn matrix. They investigated the changes in the microstructure and mechanical properties of the deformed Zn alloys were investigated. The results showed that ECAP significantly increases the elongation of the alloy but does not increase its ultimate tensile strength. Ultrafine-grain alloys have high strength, good plasticity matching, high wear resistance, and good biocompatibility [19,20]. Guo et al. [3] prepared an ultrafine crystalline Zn-Mn alloy with an average grain size of 0.35 ± 0.29 μm via multi-pass drawing. Its elongation can reach 236.2%. The Zn-0.5Mn alloy relies mainly on the second phase for grain refinement. It can achieve ultra-high plasticity after grain refinement, but its strength decreases with increasing elongation. Yang et al. [21] proved that Zn-Al and Zn-Cu alloys with ultrafine grains exhibit processing softening behavior. Although grain refinement effectively improves plastic forming properties, it can cause problems, such as process softening. During the plastic deformation of Zn alloys, the increase in deformation increases crystal defects and instability of the alloy and lowers the minimum recrystallization temperature. Process softening occurs during alloy recrystallization [22,23]. In addition, Zn-Mg alloys are less regular and show a decrease in elongation with decreasing grain size. Still, the regularity of this behavior is weak and sometimes even contrary to what is expected. Strength retention and plasticity enhancement for Zn alloys have become important research directions in biodegradable metals.

In this study, high-strength Zn-Li alloy was processed by rotary forging. A grain-refined Zn-Li alloy was then obtained, and the plasticity improvement and suppression of the processing softening of the Zn alloy were realized. This work can guide the application of Zn-Li alloys as medical biodegradable metals.

## 2. Materials and Methods

### 2.1. Materials and Processing

The Zn-0.45Li alloy used in this study was melted in an induction furnace using pure Zn (99.99 wt.%) and Li (99.99 wt.%) under Ar gas protection through the following steps. First, 85% pure Zn and Li were placed in the induction furnace and heated to 650–700 °C. After melting, the remaining Zn and Li were added and stirred continuously to mix well and remove the slag. Second, the molten alloy was held for 40 min with constant stirring, and the impurities were removed during the holding period. Third, the molten alloy was poured into a mold with a diameter of 60 mm. The ingots were shaped into blocks with 50 mm diameter and formed into bars with 11 mm diameter via hot extrusion. The hot extrusion temperature was 220 °C, and the extrusion ratio was 20:1. Lastly, the Zn-0.45Li bars were rotary-forged several times at a speed of 1 mm/s at room temperature. The deformation in each step was <18%. Coolant was used to cool the sample and increase surface lubrication during rotary forging. The solid bars obtained by rotary forging using a rotary forging machine exhibited total deformation rates of 6.2% (10.85 mm diameter), 58.1% (7.25 mm diameter), and 75.7% (5.52 mm diameter) under the extruded condition. For convenience, the 6.2%, 58.1%, and 75.7% deformation bars were labeled as R1, R2 and R3 alloys, respectively. Total deformation (Rp):

R_p_ = (S_T_ − S_a_)/S_T_ × 100% (1)

where S_T_ is the cross-sectional area of the extruded bar, and S_a_ is the cross-sectional area after rotary forging.

The alloy chemical composition was analyzed by inductively coupled plasma emission spectrometry (PE Optima 2100DV), an important method of qualitatively and quantitatively analyzing trace elements. A powder of 0.2 g ± 0.001 g was removed from the center of a Zn-0.45Li alloy bar and dissolved in 6 mL of hydrochloric acid and 2 mL of nitric acid. After the complete reaction, the powder was fixed in a 100 mL steel volumetric flask and tested to obtain the specific composition of the prepared alloy. The content of Li element in Zn-Li alloy is 0.4388 (wt.%), and the balance is Zn element.

### 2.2. Microstructural Characterization and Composition Analysis

The phases of the alloys were determined by scanning electron microscopy (SEM, FIE Sirion 200) and X-ray diffraction (XRD, BRUKER AXS). XRD was performed using CuKα radiation (λ = 1.540562 Å). The scanning speed was 0.025°/s, and the scanning range was 20–90° with a step size of 0.02°. An in-situ stretching experiment was conducted under SEM observation, and a scanning electron microscope was used to observe the change in the microstructure of the sample under stretching conditions. In addition, electron backscatter diffraction (EBSD, Nordly max3) and transmission electron microscopy (TEM, Talos F200x) were performed to examine the microstructure and morphology of the alloys. The samples used for the EBSD measurements were carefully ground on silicon carbide paper with more than 2000 mesh. EBSD samples were prepared by electrolytic polishing under Fischione 1061 ion plane polishing treatment. In particular, 6 KV was used for 1 h, followed by 4 KV for 30 min. In situ tensile samples of 14 mm length and 1 mm thickness were cut out of the alloy bars. 

In situ tensile samples of 14 mm length and 1 mm thickness were cut out of the alloy bars. The in situ stretched samples were fixed on the insitu stretching table and observed continuously by SEM. 

A TEM specimen disk with a diameter of 3 mm and a thickness of 150 μm was cut from the sample surface. Grinding was performed using 2000 mesh silicon carbide sandpaper. The sample was ground to a thickness of 30–50 μm and then ion-milled and polished with Gatan 691 at a low angle to prepare electron transparent areas on the sample. The sample was bombarded at two angles, 15° and 8°, with the voltage set to 4 and 3.5 KV, and then perforated to form the thin zone required for observation. TEM was performed at an accelerating voltage of 200 KV.

### 2.3. Measurement of Tensile Properties

The sample used to measure tensile properties was processed into a dog bone shape with a standard distance of 28.0 ± 0.1 mm and a diameter of 5 mm. Mechanical tests were performed with a desktop electronic universal testing machine (MTS 100 KN). In accordance with ISO6892-1, three experiments were conducted for each group of samples under room temperature conditions. The crosshead speed of the universal material testing machine was 1 mm/min [24]. Due to the large elongation of the alloy, an extensometer was not used in the test. Instead, SEM was used to examine the fracture morphology of the tensile specimens.

Ultimate tensile strength (UTS) is the maximum stress the material can withstand before fracturing under external force. Elongation is the percentage of the total deformation ΔL of the standard distance section after the tensile fracture of the sample to the original standard distance length L.

## 3. Results

**Figure 1:**
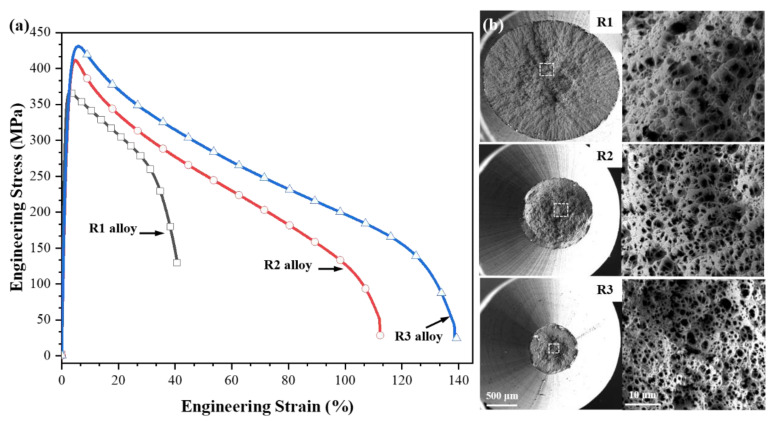
(**a**) Tensile stress-strain curves of different deformation samples and (**b**) fracture morphologies of samples.

Figure 1a shows the engineering stress–strain curves of the Zn-0.45Li alloy after several rotary forging deformations at room temperature. The mechanical properties of the Zn-0.45Li alloy were remarkably enhanced after rotary forging deformation. The maximum elongation at break of the R1 alloy was 40.6 ± 0.4%, and the ultimate tensile strength (UTS) was 380.2 ± 7.5 MPa. The elongation and tensile strength of the Zn-0.45Li alloy increased with the deformation amount. The maximum elongation of the R3 alloy was 139.2 ± 18.6%, and its UTS reached 426.1 ± 4.7 MPa. Its elongation at break increased considerably by 98.6%, and its UTS increased by 45.9 MPa. 

Figure 1b shows the tensile fracture morphology of the alloy. The fracture exhibited necking, and the fracture morphology showed a typical ductile fracture. The number of ductile nests increased gradually with the increase of rotary forging deformation. The R3 alloy had the smallest size of reduced neck and dimples.

**Figure 2:**
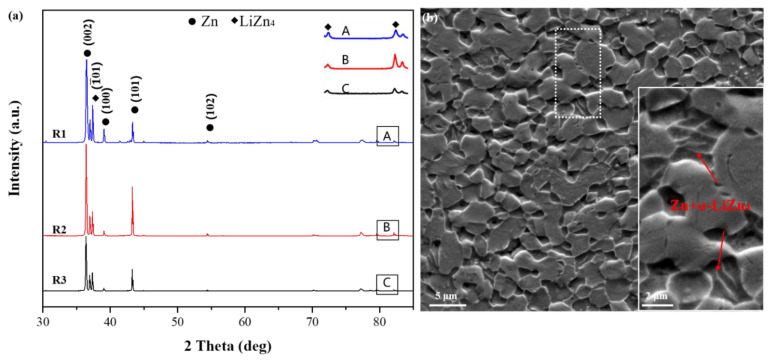
(**a**) XRD spectra of Zn-0.45Li alloys and (**b**) SEM image of the R1 alloy.

Figure 2a shows the XRD patterns of alloys R1, R2 and R3. The Zn-Li alloy had an HCP structure with a phase composition of Zn (a = 2.665 Å, c = 4.947 Å, c/a = 1.86) and LiZn_4_ (a = 2.771 Å, c = 4.379 Å, c/a = 1.58). This finding agrees with the Zn-Li phase diagram and other findings [25,26]. The diffraction intensity of (100) and (102) crystal planes of Zn grains decreased when the deformation increased. The diffraction intensity of the (101) plane increased first because of the growth of grains with this plane. Then, the diffraction intensity of the (101) plane decreased due to grain refinement. The increase in the amount of deformation did not change the content of the second phase. However, the height of the diffraction peak slightly changed, possibly due to grain refinement, grain rotation, or recrystallization during deformation. Figure 2b shows the SEM microstructure of the R1 alloy. The microstructure comprised elliptical Zn grains and needle-like α-LiZn_4_ [27,28]. This observation is consistent with the XRD results.

**Figure 3:**
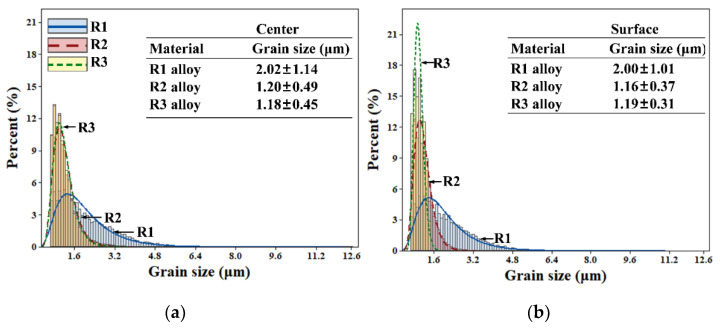
Average grain size and size distribution of R1, R2 and R3 alloy at the (**a**) center and (**b**) surface on the longitudinal section.

Figure 3 shows the grain sizes obtained by EBSD for R1, R2 and R3 alloys at the center and surfaces of the cross-section. The average grain size at the center of R1 alloy was 2.02 ± 1.13 μm, and that at the center of R3 alloy was 1.18 ± 0.45 μm. The grain size at the surfaces of these alloys decreased and then increased the deformation. As a result, the surface grain size of R3 was slightly larger than R2. The average grain size at the surface of R2 alloy was 1.16 ± 0.37 μm, and that at the surface of R3 alloy was 1.19 ± 0.31 μm. The surface grain size of R3 was slightly larger than R2, probably due to recrystallization. In general, the grain size distribution of R3 shifted to the left and tended to be narrower than those of R1 and R2, indicating that the grain size of R3 is relatively uniform. However, grain size comparison between the center and surface showed that the grain size increased first at the surface of R3 during grain refinement.

Since recrystallization consumes the stored energy, recrystallization occurs first, where the stored energy is sufficient. During deformation, deformation energy storage occurs inside the grain, placing the grain system in an unstable state. The greater the degree of deformation, the more defects form inside the metal and the more unstable the overall organization. As a result, recrystallization temperature decrease. Recrystallization occurs when the deformation amount reaches a certain level. During extrusion, the surface of the workpiece is subjected to a large external force, and its surface energy storage is large. Therefore, recrystallization occurs first at the part where the energy storage is large [29]. After recrystallization, new grains with low energy storage in the microstructure are formed, and the grains are uniform and continuous equiaxial crystallization. Hence, the grain size increases first at the surface of R3, and the grain size distribution narrows.

**Figure 4:**
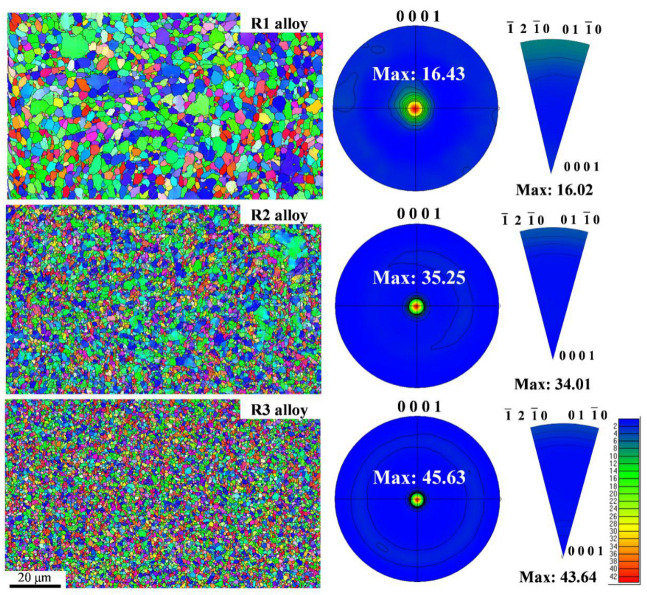
Microstructure, the (0001) pole figures (PFs), and IPF evolution at the cross-section of alloys.

Figure 4 shows the microstructure and texture evolution of R1, R2, and R3 alloys. The microstructure of the Zn-Li alloy can be visualized from the diagram, and the grain size showed a decreasing trend with increasing deformation. The grains of hot-extruded R1 alloy were not uniform, with large and small grains and elongated, deformed grains. The R2 alloy was mainly composed of small grains formed after the fracture of large grains. The R3 alloy had a uniform structure and equiaxial recrystallization of grains. As a metal with an HCP structure, the texture of Zn alloy plays a key role in its mechanical properties. According to the pole plot, the (0001) texture was dominant in all Zn-0.45Li alloy samples. The texture strength of the R1 alloy was 16.43. The texture strength of R3 increased from 16.43 to 45.63. Metal material usually forms a strong texture during plastic deformation. Texture strengthening can improve the strength of magnesium, titanium alloys and copper alloy [30,31,32,33]. Therefore, increasing texture strength after forging may enhance the strength of the Zn-0.45Li alloy.

**Figure 5:**
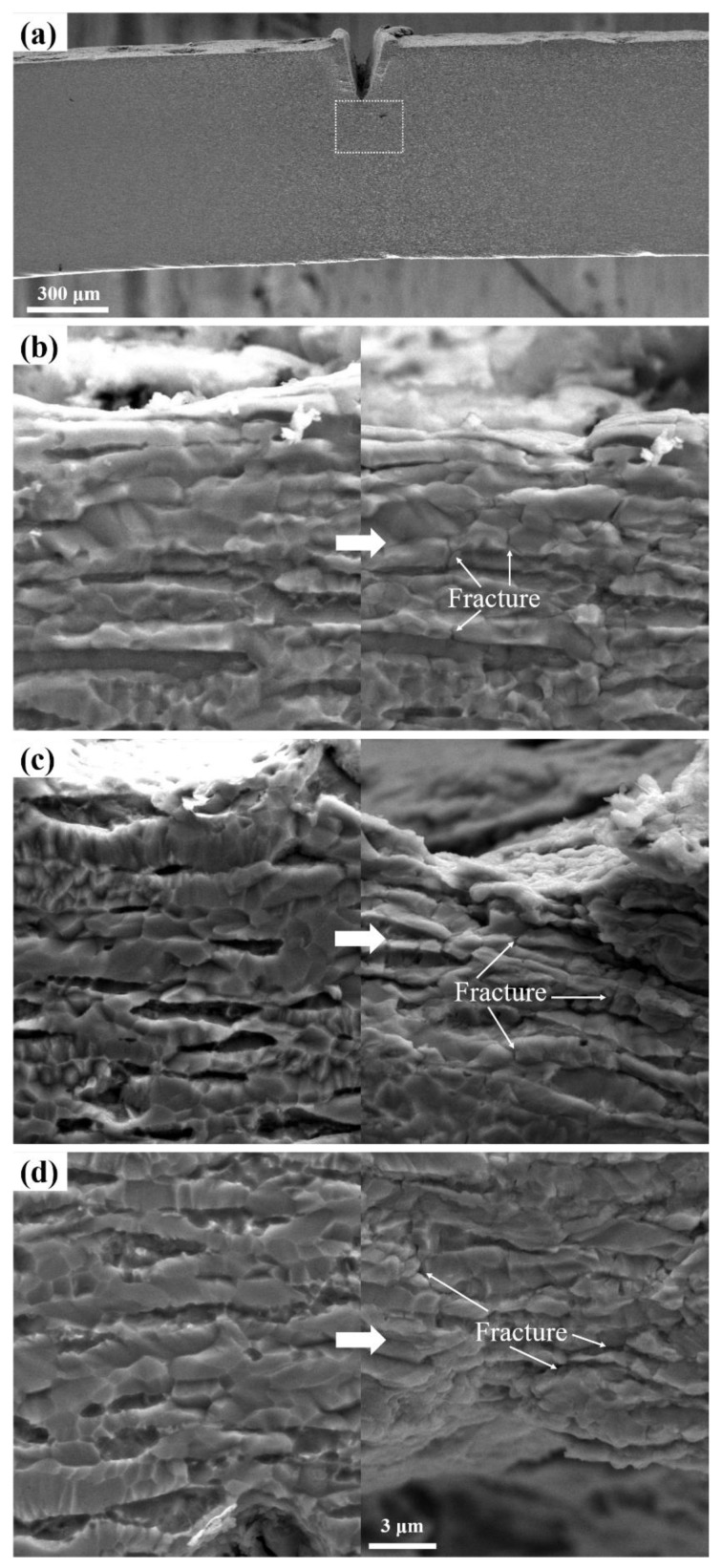
In situ tension: (**a**) observation place, (**b**) R1 alloy, (**c**) R2 alloy, and (**d**) R3 alloy.

Figure 5a shows the in situ stretching observation site. According to the in situ tensile diagram (Figure 5), R1, R2 and R3 alloys, all produced cracks along the grain boundaries when stretched in situ. The final fracture was at the grain boundary.

**Figure 6:**
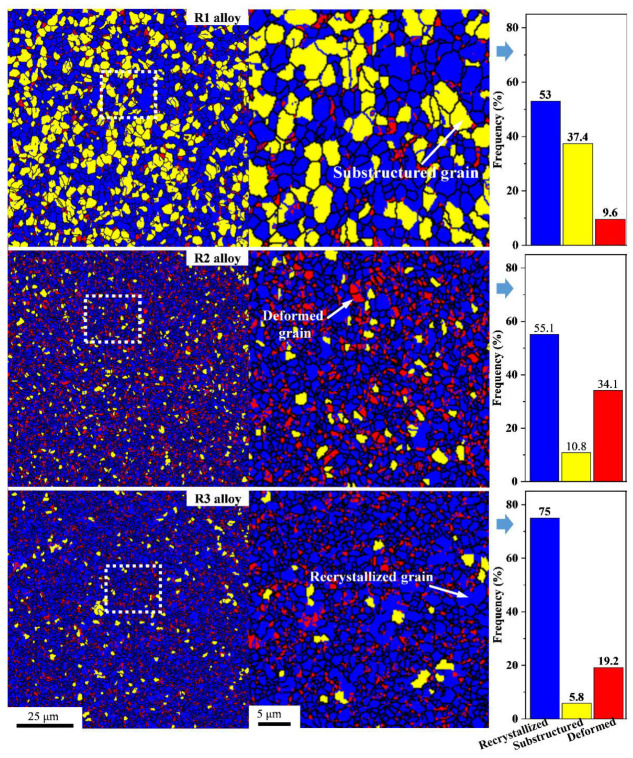
Grain boundary distribution and grain distribution composition of R1, R2, and R3 alloys.

As shown in Figure 6, recrystallized grains, substructured grains and deformed grains coexisted in the R1 alloy. With the increase in deformation, the large and sub-structured grains were broken. Hence most of the sub-structured grains in the R2 alloy disappeared, and the proportion of deformed grains increased dramatically. At this time, the proportion of recrystallized grains in the R3 alloy increased substantially, and the grains were uniform and exhibited continuous isometric crystallization (Figure 4). Recrystallization consumed the stored energy [29], so the deformed grain grew to a regular shape. The size of the recrystallized grains was highly related to the deformation degree and the size of the original grains. The greater the deformation degree, the smaller the size of the new grains after recrystallization and the more uniform their distribution [34,35]. No notable decrease in grain size was observed from R2 to R3 during rotary forging deformation, possibly due to the recrystallization that occurred during the continuous plastic deformation.

## 4. Discussion

The second phase has a key influence on grain refinement. During the grain refinement of Zn alloys, the second phase exerts a pinning effect at grain boundaries, stabilizing them and inhibiting the growth of newly refined grains [2,3]. As a result, during the plastic deformation of Zn alloys, the grain size decreases and the strength of the alloy changes. The relationship between the strength of the alloy and the grain size can be calculated from the Hall–Petch equation as follows [36]:

(2) $$\sigma_{s}=\;\sigma_{0}{+\mathrm{kd}}^{\frac{-1}{2}},$$

where σ_s_ is the alloy yield strength, σ_0_ is roughly equivalent to the yield strength of a single crystal, d is the grain diameter, and k is a constant associated with the grain boundary. According to the Hall–Petch equation, the finer the grain diameter d during plastic deformation of the alloy, the higher the yield strength σ. In addition, the plastic toughness of the material will not decrease. For most metallic materials, grain refinement can improve strength. For example, magnesium alloys, copper alloys and CoCrFeNi high entropy alloys [37,38,39]. However, the relationship between grain size and strength of Zn alloys does not exactly follow the rule. The strength of the Zn alloy increases when the manganese content in the Zn-Mn alloy is increased to refine the grain [2]. It has been found that for Zn alloys refining the extruded Zn-Mn, Zn-Cu, and Zn-Ag alloys by stretching and ECAP did not increase their strength [3,18]. When grain refinement occurs in the same area, the smaller the grains, the more grain boundaries and the higher the resistance. When the area of grain boundaries increases, they impede the movement of dislocations. Grain boundaries can limit plastic deformation to a certain extent; deformation occurs in multiple grain boundaries, so plastic deformation is homogeneous [38,40]. The grain size tends to be uniformly distributed in the surface areas of the R3 alloy, although a slight increase in the grain size is observed. As a result, the elongation of the R3 alloy is significantly increased. The main contribution of grain refinement to the Zn-Li alloy is the increase in plasticity. 

The correlation between the dislocation density and the integrated mechanical properties is consistent with the increase in alloy strength. The dislocation density is proportional to the strength and hardness of the sample [41,42]. The dislocation population generated near the grain boundaries generates a reaction force in the grain, which increases with the number of dislocations. When a certain value is reached, the dislocation source stops initiating, and the crystal strengthens significantly. The dislocation density in the material increases with plastic deformation, so we calculate the dislocations by kernel average misorientation (KAM). KAM indicates the local strain distribution in a crystalline material. KAM is a core of 24 nearest neighboring points, which assigns a scalar value to each point, indicating its local orientation difference. The KAM map obtained in EBSD can be used to calculate the geometric dislocation density and thus determine the state of the material stress distribution during the deformation process. The density of dislocations in a material can be quantified by KAM using the following equation [43,44]:

(3) $$\rho_{\mathrm{GND}}=\frac{2\Delta \delta }{\mu b},$$

where $\rho_{\mathrm{GND}}$ is the geometrically necessary dislocation (GND) density of the measured point, Δδ indicates the local orientation error, μ is the unit length of the point, and b indicates the Burgers vector whose magnitude is a constant of Zn [2]. The local orientation error is calculated as:

(4) $$\Delta \delta =\frac{1}{n}\sum_{j=1}^{n}\left|\delta_{j}-\delta_{i}\right|,$$

where $\delta_{j}$ is the local KAM value at point j, $\delta_{i}$ is the local KAM value at point i, and n represents the number of points in the test area. When the threshold angle is set to 5°, the dislocation density of R1 is calculated as 3.32 × 10^14^ m^−2^, that of R2 is 4.63 × 10^14^ m^−2^, and that of R3 is 3.64 × 10^14^ m^−2^.

**Figure 7:**
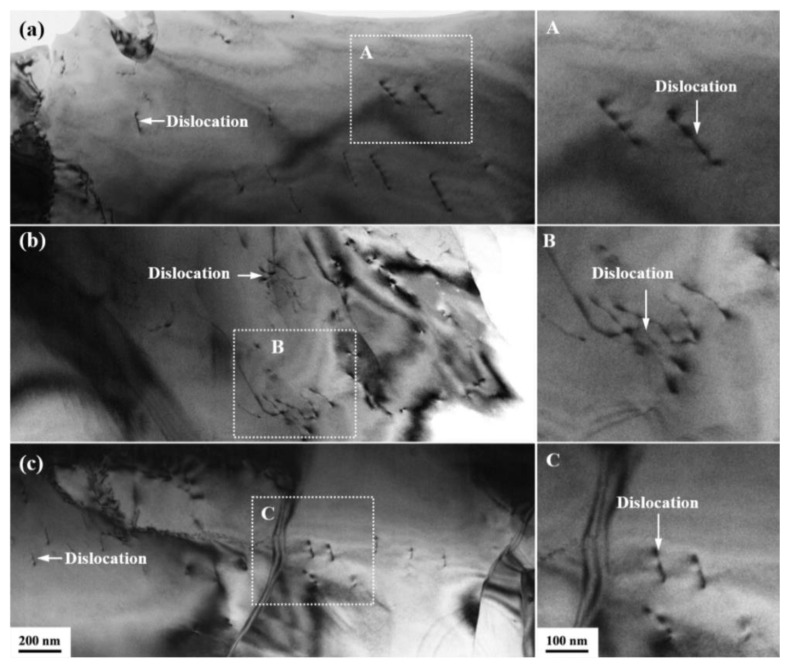
Dislocations in the TEM micrographs of (**a**) R1 alloy, (**b**) R2 alloys and (**c**) R3 alloys and **A**–**C** are local enlarged images of (**a**–**c**) respectively.

The trend of dislocation density is increasing and then decreasing, and the rate of dislocation density of R3 alloy is greater than that of R1. The significant grain refinement, accompanied by a significant increase in KAM values during the early stages of spin-forging deformation, is responsible for the increase in deformed grains and the decrease in grain size. This phenomenon indicates a significant accumulation of dislocations in the material after the spin-forging deformation of R1 to R2 alloy. The low KAM values of the spin-forged deformed samples are mainly concentrated inside the grains, and the high KAM values are mainly located at the boundaries around the grains. Thus, dislocation evolution plays a crucial role in the nucleation and growth of DRX grains by stimulating nucleation, consuming dislocations and mitigating deformation. The sub-boundaries formed by these dislocations stimulate the refinement of the original grains into deformed grains. With increasing deformation, the KAM of R3 alloy decreases compared with that of R2 because the proportion of R3 recrystallization increases. Recrystallization consumes some of the dislocations [45]. In addition, the grain size increases slightly due to the synergistic depletion effect of continuous DRXed and dynamic recovery. The dislocations are observed by TEM, as shown in Figure 7. Due to the interaction between dislocations in the alloy (stacking, entanglement), the dislocations cannot continue moving, and the deformation resistance increases [46]. As a result, the strength of the R3 alloy increases. In terms of plasticity, the effect of dislocations on the plasticity of R3 alloys is weaker than the influence of grain refinement. Therefore, the elongation of the R3 alloy maintains an increasing trend. Existing studies confirmed that the strengthening of Zn-Li alloys is a combination of fine lamellar organization consisting of Zn and LiZn_4_ and precipitation strengthening [14]. However, the present work found that the texture strength and dislocations are also enhanced. Therefore, the strength and plasticity enhancement of Zn-Li alloys after macro deformation combines dislocation strengthening, texture strengthening, grain refinement, and precipitation strengthening, rather than only fine grain strengthening, as observed in conventional macro-deformed Zn alloys.

## 5. Conclusions

This study investigated Zn-0.45Li alloys with different amounts of rotary forging deformation. As a result, the following conclusions were obtained.

(1) Grain refinement becomes highly pronounced with increasing deformation. However, the slightly larger grain size on the surface of R3 than that on the surface of R2 is due to recrystallization.
(2) The strength and ductility of the Zn-0.45Li alloy were improved simultaneously after rotary forging deformation.
(3) In all the Zn-0.45Li alloy samples, the (0001) texture is dominant. The texture strength in the (0001) direction increases deformation. Therefore, the increase in texture strength after rotary forging improves the strength of Zn-0.45Li alloy.
(4) The strength and plasticity enhancement of Zn-Li alloys after macro deformation was a combination of dislocation strengthening, weave strengthening, and grain refinement rather than only fine grain strengthening as observed in conventional macro deformed Zn alloys.

## Data Availability

Data are available on request from the corresponding author.
